# Skeletal muscle proteomics: considerations and opportunities

**DOI:** 10.1038/s44324-025-00073-2

**Published:** 2025-07-02

**Authors:** Julian P. H. Wong, Yaan-Kit Ng, Jeppe Kjærgaard, Ronnie Blazev, Atul S. Deshmukh, Benjamin L. Parker

**Affiliations:** 1 Department of Anatomy and Physiology, The University of Melbourne, Melbourne, VIC Australia; 2 Novo Nordisk Foundation Center for Basic Metabolic Research, University of Copenhagen, Copenhagen, Denmark

**Keywords:** Biochemistry, Physiology

## Abstract

Skeletal muscle accounts for 30–40% of body weight and plays an indispensable role in maintaining movement and is also a central regulator of whole-body metabolism. As such, understanding the molecular mechanisms of skeletal muscle health and disease is vital. Proteomics has been revolutionized in recent years and provided new insights into skeletal muscle. In this review, we first highlight important considerations unique to the field which make skeletal muscle one of the most challenging tissues to analyse by mass spectrometry. We then highlight recent advances using the latest case studies and how this has allowed coverage of the skeletal muscle temporal, fibre type and stem cells proteome. We also discuss how exercise and metabolic dysfunction can remodel the muscle proteome. Finally, we discuss the future directions of the field and how they can be best leveraged to increase understanding of human biology.

## Introduction

Proteins underlie the ability to respond dynamically to the challenges and changing cellular environments of life. Through their various functions, modes of regulation, and complex signalling networks, proteins within cells/tissues (proteome) form the very foundation of biology. Mass spectrometry (MS)-based proteomics is a technology-driven approach for the characterisation of the proteome^1^. Over the last two-three decades, its accurate, unbiased and high-throughput nature has revolutionised the ability to understand biological mechanisms (functional proteomics) at a systems-wide scale. This has allowed the characterisation of both canonical and novel proteins and their accompanying transduction pathways important for physiology, health, and disease^2^.

Skeletal muscles account for 30–40% of total body weight and functions as the principal contractile organ of the body by generating power and force^3^. Skeletal muscle is a highly heterogeneous tissue consisting of various cell types including myofibers, endothelial cells, fibro-adipogenic progenitors, muscle stem cells, and other mononuclear cells such as resident or infiltrating immune cells. Skeletal muscle myofibers are the most abundant and are typically characterised into either slow oxidative (type I) or fast glycolytic (type II) fibres, where they play an indispensable role in maintaining posture, movement and functional independence^4^. Skeletal muscle is also an active endocrine organ capable of secreting circulatory factors (myokines) that regulate inter tissue crosstalk^5^ and has an extraordinary capacity for regeneration^6^. Furthermore, skeletal muscles are central regulators of whole-body metabolism by serving as primary sites of glucose storage and uptake in response to insulin ( ~ 80% post-prandial)^7^. Nonetheless, in states of metabolic dysfunction, skeletal muscles reduce their sensitivity to insulin and ability to uptake glucose (insulin resistance)^8^. In turn, the continued progression of insulin resistance, hyperinsulinemia and hyperglycemia can lead to severe comorbidities such as cardiovascular disease^9,10^. Together, these conditions represent one of the most common causes of burden and disability worldwide^11^.

Exercise is one of the most effective ways to promote muscle health and combat insulin resistance. Research efforts over the last two decades have collectively pointed towards its ability to increase insulin sensitivity, stimulate muscle synthesis and increase muscle strength/function depending on the type of exercise modality^12–14^. Nevertheless, exercise alone is not sufficient to prevent these functional declines, nor are there any currently approved therapeutics that specifically target skeletal muscles^15^. As such, understanding the signalling pathway underlying these conditions is vital for identifying novel therapeutic targets to improve musculoskeletal health and longevity.

Indeed, over the last two-three decades, MS-based proteomics have been used to better understand how skeletal muscle signal transduction is regulated under various settings of health and disease. In this review, we aim not to discuss the techniques nor procedures used for the preparation and analysis of skeletal muscles by proteomics, in which readers are directed to comprehensive reviews by Dowling and colleagues^16^ and Cervone and colleagues^17^. Instead, we discuss the technical challenges unique to the field and how they can be overcome to allow coverage of the spatial distribution, fibre-type differences, and MuSC of the skeletal muscle proteome. We also critically evaluate the individual merits of these approaches using the latest case studies. We describe how recent advances in skeletal muscle proteomics has increased the understanding of the molecular responses to exercise and in diseases, with a keen focus on metabolism and insulin resistance. Throughout the review, we present current limitations, highlight overlooked shortcomings, and discuss future opportunities in the field.

### Challenges of skeletal muscle proteomics

**Fig. 1:**
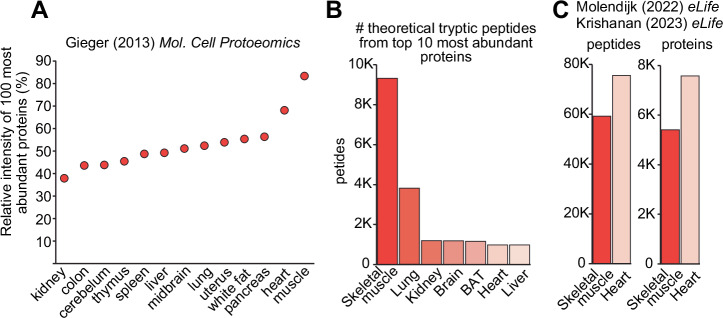
Characteristics of analysing the skeletal muscle proteome by mass spectrometry. **A** Relative intensity of the top 100 most abundant proteins in various mouse tissues. **B** Number of theoretical tryptic peptides generated from top 10 most abundant proteins in various mouse tissues. **C** Number of observed tryptic peptides in mouse skeletal muscle and heart. Figure (**A**) adapted from^18^. **C** adapted from^20,21^.

One of the major challenges in the proteomic analysis of skeletal muscles is the exceptionally high dynamic range, combined with the presence of large and abundant contractile proteins. These proteins generate thousands of peptides from tryptic digestion which saturate mass spectrometric detection of peptides from low abundance proteins. Geiger et al., explored the proteome of 28 mouse tissues revealing that the top 100 most abundant proteins in skeletal muscle contribute to more than 85% of the proteome, while in organs such as the kidney, they contributed to <40% of the proteome^18^ (Fig. 1A). In fact, comparative proteomic analysis of mouse skeletal muscle and C2C12 myotubes revealed that the top 10 most abundant skeletal muscle proteins collectively make up over 50% of total protein mass^19^.

To further demonstrate how large and high abundant proteins can hypothetically influence protein detection, we calculated the number of theoretical tryptic peptides produced from the top 10 most abundant proteins in mouse skeletal muscle compared to various other tissues. Figure 1B shows that the top 10 most abundant proteins in skeletal muscle generate almost 10 times more theoretical tryptic peptides compared to other tissues with the exception of the lung. Interestingly, while heart tissues also contain large and abundant contractile proteins, many of these (i.e., titin, myosin, and nebulin) are expressed at lower levels compared to skeletal muscles. Instead, the top 10 most abundant heart proteins consist of many mitochondrial proteins such as ATP5A1 and ATP5B, which produce fewer tryptic peptides. We re-analysed our previously published heart and skeletal muscle proteomics data from identical mice where samples were prepared in parallel, peptides fractionated into the same 12 fractions, analysed on the identical LC-MS/MS system, and processed with the identical proteome database/search algorithm pipeline^20,21^. We identified a total of 59,187 peptides spanning 5407 proteins from mouse skeletal muscles while 75,513 peptides spanning 7578 proteins were identified from heart tissues (Fig. 1C). A total of 4,664 peptides (7.9%) were identified in skeletal muscle from the large and abundant contractile proteins Titins and Myosins, while only 3583 peptides (4.7%) were identified from these same proteins in the heart. Taken together, the complexity, size and abundance of the skeletal muscle proteome makes deep proteome coverage extremely challenging.

### Separation and fractionation techniques for skeletal muscle proteomics

Comprehensive coverage of the skeletal muscle proteome requires protein and/or peptide level separation and fractionation. Early analysis of the skeletal muscle proteome of mouse gastrocnemius^22^, rat abdominal^23^, and human vastus lateralis^24^ were separated using a 2D gel electrophoresis approach, yielding 71, 74, and 107 protein identifications using matrix-assisted laser desorption/ionization-MS (MALDI-MS), respectively. Expectedly, most of these proteins were of myofibiliar and sarcomeric origins.

The introduction of more advanced liquid chromatography separation coupled to electrospray ionization and tandem mass spectrometry has propelled the field forward enormously, with Højlund et al. leveraging the high resolution Fourier Transform Ion Cyclotron Resonance MS to identify 954 unique protein groups in the vastus lateralis muscle in healthy men, revealing canonical proteins involved in the myofibrillar apparatus, calcium homeostasis and glucose, glycogen and lipid metabolism^25^. The authors detected 212 mitochondrial proteins which accounted for ~22% of the skeletal muscle proteome, compared to the expected 4.8% found in the total human proteome^26^.

In 2015, one of the deepest skeletal muscle proteome analyses was performed using isoelectric focusing-based fractionation of peptides derived from C57BL/6 J mouse triceps paired with C2C12 myotubes^19^. This allowed peptides identified in the C2C12 myotubes to be matched to peptides identified in the mouse skeletal muscles based on MS1 features and peptide retention time (commonly referred to as “Match Between Runs” [MBR]). This approach led to identification of 10,218 proteins across the model systems (C2C12: 9880 and muscle: 8309 proteins) whereas when authors analysed raw files from skeletal muscle and C2C12 separately, 30% less proteins were detected in skeletal muscle (5887 proteins). While MBR is incredibly powerful and able to significantly boost peptide identification, it may not be suitable for every application and without additional filtering, false positive identifications have been documented^27^. Nevertheless, this strategy combined with peptide level fractionation enabled quantification of low abundance proteins such as transcription factors—MYOD1, MYOG, MEF2C, nuclear receptors—SMAD1 and NOTCH3, and circadian clock proteins—BMAL, CRY1 and FBXL3. These authors also provided an estimation of the absolute abundance of the mouse skeletal muscle proteome using Intensity-based absolute quantification (iBAQ)^28^, which to date, still represents an invaluable resource to the field.

In addition to protein/peptide level fractionation at a whole muscle level, subcellular fractionation of skeletal muscle is another attractive approach to obtain greater proteome coverage of discrete organelles. Considering the importance and abundance of mitochondrial proteins, it is unsurprising that a majority of subcellular skeletal muscle proteomics studies have focused on the mitochondria. Lefort et al. employed a differential centrifugation approach to specifically enrich mitochondria in human vastus lateralis muscles^29^, revealing the identification of 487 mitochondrial proteins including canonical proteins involved in the electron transport chain (ETC), TCA (tricarboxylic acid) cycle, fatty acid oxidation and mitochondrial transporters. The authors also identified 9 out of the 13 mtDNA (mitochondrial DNA)-encoded proteins. Adelnia et al. then extended this investigation by performing an integrated proteomics analysis of mitochondrial energy capacity in 57 healthy adults (22 F and 35 M)^19^. The authors assessed in vivo mitochondrial function (31 ^P^ magnetic resonance spectroscopy) paired with LC-MS/MS of the vastus lateralis muscle, revealing 4300 proteins, for which 253 were associated with increased and 93 proteins with decreased oxidative capacity, respectively. Enrichment analysis revealed that the 30% of the proteins positively associated with energetics were localised in the mitochondria with many of the proteins being involved in complex I, ETC, fatty acid metabolism, and critic acid cycle. Since these initial investigations, several additional studies have applied similar proteomic approaches to study skeletal muscle mitochondrial changes in Duchenne muscular dystrophy (DMD)^30^, exercise training^31–34^, ageing^35^ and insulin sensitivity^36^. In addition to mitochondrial isolations, Martin et al. performed sucrose-based fractionation to isolate nuclei and identified 854 nuclear enriched proteins with an enhanced coverage of proteins associated with DNA/chromatin-binding compared to analyses of whole cell lysate (WCL)^37^.

Biochemical-based fractionation is another common approach to increase depth of coverage by separating classes of proteins with different physico-chemical properties. Recently, Roberts et al., separated human *vastus lateralis* muscle biopsies into myofibril (MyoF) and non-myofibril fractions utilising a corona nanoparticle strategy^38^ to identify approximately 5645 non-MyoF and 2611 MyoF proteins identified in each individual^39^. This approach uses multiple nanoparticles with distinct physicochemical properties that bind specific proteins. However, it should be noted that the authors did not report on the precision or accuracy of the approach. Moreover, a comprehensive analysis of how different lysate compositions influence protein capture on the nanoparticle has, to our knowledge, not been published for skeletal muscle. Martinez-Val et al. established a spatial proteomic approach using biochemical fraction and identified discrete changes in the subcellular distribution of ribosome subunits following electrical stimulation of mouse tibialis anterior (TA) muscles^40^. Finally, an additional spatial proteomics approach was recently introduced by Schmidt et al., which combined serial cryosectioning of mouse soleus muscle with proteomics to characterise the myotendinous junction (MTJ) proteome^41^. Using zonation-based clustering, the authors elegantly profiled the muscle-tendon transition. Many of the enriched proteins were also identified by serial extraction of semitendinosus muscle-tendon samples with detergents of increasing stringency^42^.

**Fig. 2:**
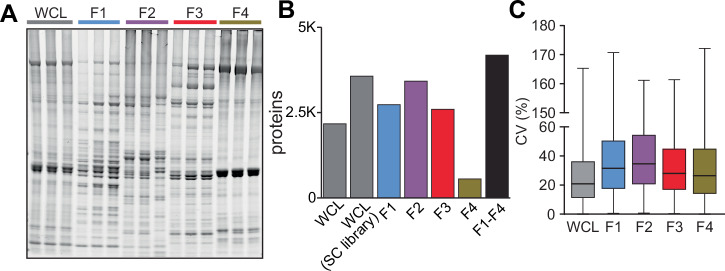
Comparison of the proteomic analysis of whole cell lysate verses biochemical subcellular fractionation. **A** Total protein stain by SyproRuby of mouse skeletal muscle whole cell lysate (WCL) and four subcellular fractions. **B** Number of proteins identified in WCL and subcellular fractions by single shot LC-MS/MS with DIA. **C** Coefficient of variation of the proteins identified in all 3 technical replicates of the WCL and subcellular fractions.

While these various fractionation approaches have expanded the coverage and understanding of the skeletal muscle proteome, the extended upstream separation and handling steps during sample preparation will increase technical variation and may negatively influence statistical power to detect biological changes during quantitative experiments. To evaluate this, we generated new data to compare the technical variance and proteome depth when analysing whole skeletal muscle extracted with harsh denaturing conditions (whole cell lysate [WCL]) versus a more gentle biochemical subcellular fractionation (SC) approach. A single mouse *gastrocnemius* muscle was powdered under liquid nitrogen and aliquoted in 6 equal amounts to rule out any biological or spatial variation. Three aliquots were extracted in harsh 6 M guanidine via repeated tip-probe sonication and heating at 95 °C representing the WCL. The remaining three aliquots were extracted with gentle mechanical lysis using (Omni International; TH-02) and four fractions generated with the Thermo Scientific Subcellular Fractionation Kit using progressively increased detergent concentration to extract organelles/proteins. SDS-PAGE and total protein staining revealed overall good fractionation with many protein bands unique to specific fractions (Fig. 2A). Analysis by single-shot LC-MS/MS using data-independent acquisition (DIA) identified a total of 2167 proteins in the WCL while 4185 in the combined four subcellular fractions (Fig. 2B). Using the SC fractions as a spectral library for the analysis of the WCL increased the number of identified proteins to 3563. The median coefficient of variation (CV) in the WCL was 20.9% which significantly increased to 31.5%, 34.6%, 28.0, and 26.4% in the SC fractions F1-F4, respectively (Fig. 2C). These data demonstrate that fractionation can increase proteome depth but often comes at the cost of increased technical variation and time. The decision to take this penalty during quantitative experiments will ultimately depend on the biological variation, intervention effect size and cohort size/replicate numbers.

In summary, subcellular or biochemical fractionation of the skeletal muscle proteome is an extremely important approach to increase coverage depth or to characterise the spatial distribution of distinct cellular compartment/cell types. Nonetheless, under some experimental conditions, fractionation may increase technical variation and negatively impact statistical power. While advances in fractionation and separation techniques coupled to analysis with next generation mass spectrometers has enabled deeper coverage, rapid and comprehensive identification of the skeletal muscle proteome remains incomplete. For example, Lai et al., have identified >9200 proteins from non-fractionated brain tissue^43^, and several groups have recently identified >10,000 proteins from non-fractionated human cells lines in <1 h of acquisition time^44,45^.

### Fibre type specific proteome

Skeletal muscles contain a heterogenous mix of fibre types, which can be broadly characterised as slow-twitch (type I) or fast-twitch (type IIA, IIX/D, and IIB) depending on Its myosin heavy chain (MHC) expression^4^. Rodent skeletal muscles are known to contain the full spectrum of fibre types, whereas human skeletal muscles only contain type I, IIA, and IIX/D fibres.

Classically, type I fibres are characterised by the presence of slow-twitch MHC I (encoded by the MYH7 gene) and typically contract slowly (slow speed of shortening) but have a high oxidative capacity (from oxidative phosphorylation) to support prolonged energy utilisation (thus, these fibres are relatively fatigue resistant). Type II fibres such as IIA, IIX/D, and IIB, are characterised by the presence of MHCIIa (encoded by MYH2), MHCIIx/d (encoded by MYH1) and MHCIIb (encoded by MYH4), respectively. They generally contract faster (IIA < IIX/D < IIB) but are more fatigable (IIA < IIX/D < IIB) than type I fibres, with IIA fibres utilising a combination of oxidative and glycolytic metabolism for fuel generation, while IIX/D and IIB fibres prioritises glycolytic metabolism^46,47^. For example, the mouse fast-twitch extensor digitorum longus (EDL) muscle is known to be composed of mainly type II fibres ( > 90%) while mouse slow-twitch soleus muscle is composed of ~60% type II and ~40% type I fibres^48–50^. In practice, muscles often exist as a spectrum of fibre types rather than comprising of pure fibres only and generally follow a sequential fibre-type transition pattern I ↔ IIA ↔ IIX/D ↔ IIB^51^. Today, the use of mass spectrometry-based proteomics has enabled a deep exploration of how the structural, metabolic and functional profile of muscles can differ depending on their fibre type composition.

Drexler et al., investigated these differences by characterising the proteome of the soleus (generally more oxidative) and EDL muscles (generally more glycolytic) in the SILAC mice, revealing 2,163 shared and 551 regulated protein groups between the two^52^. The authors identified canonical proteins involved in Ca^2+^ handling, metabolism and contractility that differ between the two skeletal muscle types. Whilst whole muscle analysis of slow and fast twitch muscles can reveal differences in their proteomes^53^, the existence of mix fibre type composition can affect the interpretation of more subtle phenotypes. To tackle this, Murgia et al., employed single fibre proteomics label free quantification (LFQ) to characterise proteomic differences in type I, IIA, IIX/D and IIB mouse muscle fibres, stratified by fibre typing based on the presence of MYH isoforms. Through their integration of single fibre with whole muscle proteomics in the MaxQuant environment and MBR, they were able to quantify almost 3000 proteins per single fiber^54^. The authors managed to characterise canonical proteins such as Myomesin 3, which is upregulated in I and IIA but not IIX/D or IIB fibres, and novel proteins including STAC3—a protein involved in excitation-contraction coupling which was upregulated in IIX/D and IIB, and Mitsugumin-53/Trim72—a protein involved in membrane repair and was upregulated in type I fibres. Moreover, the authors reveal an unexpected degree of mitochondrial specialisation with the major mitochondrial pathways—OXPHOS, beta oxidation and the TCA cycle all showing significant variation in different fibre types^55^. The same group then took this one step further using a similar experimental approach to investigate the human muscle fibre proteome, revealing 3852 unique protein groups with 404 differentially regulated proteins between fibre types^56^. Many of these proteins show significant differences in abundance across the organisms and confirm that the fibers richest in mitochondria are the slow type I fibers in humans and the type IIA fibers in rodents.

While early single-fiber proteomics studies focused on freshly isolated fibers from rodents and humans, recent advancements, including optimized workflows utilizing freeze-dried samples, have expanded research possibilities by allowing the use of biobank samples from previous studies. For example, Deshmukh et al. profiled isolated single muscle fibres from rapidly snap frozen, freeze-dried human muscle biopsies^57^, coupled to proteomic analysis of primary human cells using LFQ, revealing 3360 unique proteins groups and 471 regulated proteins between fibre types. The same research group extended the analysis to the largest single muscle fiber proteome study where >1000 single muscle fibres were analysed^58^. They utilized highly sensitive instrumentation combined with a short 21-min chromatographic gradient, achieving impressive coverage of nearly 3000 proteins. This efficient workflow allows for the analysis of up to 60 single fibers daily with high reproducibility and depth. It revealed substantial heterogeneity within each fiber type and demonstrated that expression of MYH isoforms alone does not define muscle fibre’s molecular profile as variations in metabolic, ribosomal, and cell junction proteins also contribute significantly to overall diversity. These findings highlight the unique insights afforded by single-fiber analyses. It is also important to note that the use of different protein quantification algorithms can significantly affect interpretation of results. Momenzadeh et al., [PMID: 37463334], analyzed 53 human single fibers by either iBAQ [PMID: 21593866] or by MaxLFQ [PMID: 24942700], revealing that iBAQ quantification was more reliable than MaxLFQ in assigning MYH isoforms based on established literature. For example, expression of MYH4, a specialized isoform found in rodents but are present in negligible quantity in humans, was 0.3% of total MYHs when using IBAQ, compared to 10% of total MYH when using MaxLFQ.

Nonetheless, an important limitation of the above fibre-type specific proteomic analyses is that stratification was performed primarily on myosin isoform expression and no phenotypic assessment was performed on individual fibres. To overcome this limitation, Ng et al. functionally characterised calcium handling and contractile properties of mechanically skinned single fibres combined with paired proteomic analysis of the same fibre^59^. An advantage of this approach is rather than stratifying fibres based on myosin expression, the authors grouped fibers based on functional parameters and correlation analysis was used to associate these functional phenotypes to the abundance of 1612 proteins in a pairwise manner. For example, fibre-type specific sensitivity of the contractile apparatus to calcium is mediated by the expression of Troponin C isoforms which each bind a different number of calcium ions, and hence an expected positive correlation of the slow troponin C isoform (TNNC1) and a negative correlation of the fast troponin C (TNNC2) with the concentration of calcium needed to elicit 50% of maximal force (also known as the pCa50) was observed. In addition to these known associations, hundreds of additional novel associations were identified such as a positive correlation between ribosomal protein subunits and specific force. An important limitation to the analysis performed by Ng et al. is that phenotypic analysis of the contractile apparatus and calcium handling was performed on mechanically skinned fibers prior to proteomics analysis. This involves removal of the sarcolemma and the authors show that this results in loss of ~22% of the proteome compared to analysis of intact fibers. In a similar analysis, Seaborne et al. performed a paired proteomic analysis of myofibers isolated from human patients with nemaline myopathy coupled with analysis of the myosin super-relaxation state (SRX)^60^. Correlation analysis identified several associations including proteins with ATPase activity which is important given the role of myosin SRX and skeletal muscle energy expenditure. While this analysis provides new insights into potential functional roles of fibre-type protein expression and phenotypic differences, it is important to note that these associations may not be causal in nature, and both studies have not performed additional validation studies.

On the whole, proteomic analysis, especially at a single fibre level have provided novel insights into protein expression changes across fibre-types and how they change during interventions such as exercise. However, current single fiber proteomic analysis is still limited to the detection of ~3000 proteins. Looking ahead, it is important that the field further enhances methodology to improve sensitivity and mechanistically explore if these proteins contribute to the various contractile and/or metabolic differences between fibre-types. For example, research should now focus on genetic studies that over-express (e.g using fiber-type specific promotors) or knockout these proteins in specific fibre-types and assess their functional role at either the whole muscle- or single fibre-level in settings of health and/or disease.

### Muscle stem cells

Skeletal muscle stem cells (MuSCs) or satellite cells, are the resident stem cells of the skeletal muscles, responsible for homeostatic regulation and regeneration following injury^61^. In the face of injury, quiescent satellite cells rapidly activate and re-enter the cell cycle to start proliferating, with some daughter cells differentiating to form myoblasts, and others returning to quiescence to replenish the stem cell pool. Myoblast then terminally differentiate and fuse to form multinucleated myofibres^62^. This section will primarily focus on studies that investigated the stem cell proteome in adult skeletal muscles rather than during development.

Zhang et al. presented one of the first characterisations of the skeletal muscle stem cell proteome. The authors utilized FACS to isolate GFP labelled MuSCs, revealing 441 MuSC exclusive proteins by 1D-gel electrophoresis, including canonical markers such as CD34, integrin α7, caveolin-1, Numb and β1-integrin^63^. The authors identified Protein Arginine Methyltransferase 5 (PRMT5), which upon MuSC-specific KO, resulted in the complete abolishment of muscle regeneration 7, 14 days and 4 months after injury in adults. Nonetheless, these results were not seen when KO was applied under an embryonic setting, which challenges the conventional view that embryonic and adult myogenesis are largely regulated by the same molecular cues. In 2021, Schüler et al. preformed one of the largest characterisations of the mouse MuSC by quantifying >4500 proteins across 3 age groups (3 [young], 18 [old] and 26 [geriatric] months) by single-shot label-free DIA^64^. Subsequent integration with whole-muscle proteomics revealed age-dependent changes in the extracellular matrix (ECM) and dysregulation of MuSC ECM proteins, including Lrp1, Egfr, Cd44 and Smoc2 of the integrin pathway. Nonetheless, a limitation of these studies is that MuSCs were isolated by first dissociating the tissue via enzymatic digestion, followed by FACS - and this process is known to activate quiescent MuSCs. Given the rapid activation of MuSC in response to stress/injury, capturing the non-activated state was performed by, Zeng et al., using an in vivo paraformaldehyde (PFA) fixing to preserve the bona fide quiescence proteome^65^. Central to this approach is a de-crosslinking step which is typically performed in conditions of high detergents and at elevated temperature. Resultantly, the authors characterised over ~3000 proteins, including CPEB1, which resulted in impaired activation (via decreases in MyoD—a marker of activation) and proliferation (via decreased Edu uptake—a marker of cycling) upon knockdown. Together, the authors reveal the novel role of CPEB1 as a regulator of stem cell activation. Overall, this approach holds great promise, however, the additional purification steps can result in increased technical variability and decreased statistical power as discussed.

Despite being a relatively new field, great strides have been made in characterising the proteins and pathways important for MuSC function via mass spectrometry-based proteomics. We anticipate that the combination of in vivo fixation combined with recent improvements in sample preparation, such as SP3^66^ and others, will improve recovery of the limited protein amounts recovered from fixed FACS sorted MuSCs. Furthermore, the use of alternative detergents and/or solvents such as the MS-compatible surfactant - n-Dodecyl-β-D-Maltoside (DDM) also warrants further investigation^67^. In the future, the combination of improved sample recovery and miniaturised sample preparation^68^ will likely expand MuSC analysis to enable the quantification of low abundant PTMs such as phosphorylation. However, it is important to note that while PFA crosslinking can fix and preserve in vivo protein abundance over the time course of several minutes, whether this can preserve protein phosphorylation which can be recycled over a time frame of seconds, warrants further comprehensive analysis. Furthermore, cross-linking and de-crosslinking reaction times and conditions should be assessed to investigate potential artefacts and how these steps can influence endogenous quantification. Finally, investigating how the proteomes of non-satellite MuSCs, such as mesenchymal stem cells, remodel to maintain skeletal muscle homeostasis, would be valuable^69^. Overall, elucidating the proteins, their modifications and signal transduction pathways that regulate MuSC activation in vivo holds great promise to understand the regenerative capacity of skeletal muscles.

## Skeletal muscle proteomics in exercise

Physical activity is one of the most powerful modulators of health, classically categorised as either endurance (i.e., low load—high repetitions; to increase aerobic capacity) or resistance (i.e., high load—low repetitions; to induce muscle hypertrophy)^70^. Typically, endurance exercise induces adaptations in mitochondrial biogenesis/respiration rate, whereas strength training increases the synthesis of contractile proteins resulting in greater muscle size, and type I to II transition^71^. The use of mass spectrometry-based proteomics has allowed a greater understanding of the multifaceted and complex signal transduction pathways contributing to these molecular adaptations^17,72^.

Early exercise training studies in rat^73–75^ and humans^76–78^ showed that despite quantifying only a tiny subset of the proteome, a large relative proportion was remodelled. Since these studies, several additional analyses of exercise training have been performed and the establishment of the Molecular Transducers of Physical Activity Consortium (MoTrPAC) have provided comprehensive temporal maps across various biological layers, tissues, sexes, species and exercise modalities. The most comprehensive analysis of exercise training in rats was recently presented and included a proteomic analysis across seven tissues integrated with several different multi-omic analysis^79^. Proteomics was performed on the gastrocnemius muscle, collected 48 h after the last exercise bout, in rats trained for 1, 2, 4 and 8 weeks of exercise in both sexes. Six batches of TMT 11-plex, which are isobaric tandem mass tags that enable sample multiplexing to increase proteomic depth and throughput, each including a pooled internal reference to normalise across the batches. The analysis also included an enrichment of phosphorylated and lysine acetylated peptides. Overall, the analysis revealed that exercise resulted in the regulation of ~700 proteins out of the ~6000 quantified in skeletal muscles (14%). Interestingly, similar relative changes were also observed in the liver where approximately 1000 proteins were regulated out of ~8000 quantified (12.5%), as well as in the heart and subcutaneous adipose tissue, highlighting the massive systemic effects of exercise training throughout the body. This vast amount of data provides an exceptional resource for the community and can be explored via https://motrpac-data.org/.

To understand how changes in signaling following an acute bout of exercise may contribute to these long-term adaptations, several studies have performed detailed phosphoproteomics following a single exercise modality in rodents^80–82^ and humans^83–85^. Nonetheless, different exercise modalities can have profound differences in their cellular responses, which are undoubtably driven by both common and divergent complex signaling pathways. To investigate these, Blazev et al. compared the signaling responses to an acute bout of endurance, resistance and sprint exercise in humans^86^. Skeletal muscle biopsies were taken before, immediately after exercise and 3 h into recovery. Each participant completed all three exercise modalities in a randomized cross-over design following washout periods resulting in the collection of nine biopsies/participant, allowing for a powerful paired analysis. The nine samples/participant were labelled in a single batch with TMT 10-plex with the 10^th^ channel used for a pooled internal reference to normalise between the batches. This resulted in the identification of >18,000 phosphosites and >9000 phosphosites identified in all nine biopsies from 3 or more participants. However, only ~2000 phosphosites were quantified in all eight subjects (72 biopsies) highlighting current limitations for comprehensive quantification. Despite this, the >9000 phosphosites quantified displayed robust precision owing to the combination of multiplexed analysis in the same batch and the paired experimental design. This identified >5000 phosphosites regulated either immediately after or in recovery from at least one of the exercise modalities. However, the study only prioritised analysis of the signalling events common to all exercise modalities, and focused on the in vivo validation of a single phosphosite of S67 on C18ORF25 (ARKN2). Embryonic loss of *C18Orf25* reduces muscle cross sectional area, skeletal muscle contractile function and exercise capacity in mice, and phospho-mimetic re-expression in vivo increases contractile function. Interestingly, an intronic rs6507691 C > T SNP in *C18ORF25* is associated with increased *C18Orf25* expression and greater cross-sectional area in humans^87^. The T allelic frequency is ~20–30% across the general population of the 1000Genome and TOPMED cohorts whereas analysis of >200 world-class strength athletes and wrestlers revealed significantly higher frequences of 35–40%.

Taken together, these various extensive proteomic studies of exercise responses in skeletal muscle have likely mapped the majority of regulated pathways, or at least pinpointed the most abundant changes. While limitations in proteome coverage remains a hurdle, only a tiny fraction of the phosphoproteome has been functionally validated. It remains unclear the kinases and phosphatases that are responsible for the vast majority of regulated phosphosites nor how phosphorylation events drive proteome remodelling during repeated exercise bouts. The challenge of the field is to now pinpoint which of these signalling events, and identify the precise molecular mechanisms, that are critical for exercise adaptations and proteome remodelling. We anticipate mutational studies incorporating functional genomics will be a critical tool to elucidate this in a high-throughput manner, including the development of CRISPR-based screens combined with functional readouts of exercise adaptations.

## Proteomics in metabolic signalling

Skeletal muscle metabolism is essential for maintaining whole-body health. It is the principal organ responsible for glucose uptake ( ~ 80%) to enable effective glucose clearance from the bloodstream, post prandial^88^. Consequently, many metabolic diseases are intimately linked to skeletal muscle dysfunction, including type-2 diabetes (T2D)^89^, mitochondrial myopathies^90^ and glycogen storage diseases^91^. The use of MS-proteomics has since allowed a better understanding of how metabolic dysfunction remodels protein abundance and signalling networks in skeletal muscles, which holds great promise for the discovery of novel treatment avenues.

Early proteomics studies of skeletal muscle in insulin resistance states revealed alterations in structural proteins and metabolic proteins^92,93^. These findings were similarly identified in more recent studies by single-shot DIA^94^, revealing impairments in mitochondrial oxidative phosphorylation and glycolytic compensation. Together, these studies reinforce how metabolic dysfunction drives changes in the skeletal muscle proteomic landscape. Nonetheless, these earlier studies are still limited in their overall depth of analysis, with Ohman et al. quantifying only a limited ~2000 proteins (for a review see^95^).

Recently, Kleinert et al. characterised how insulin resistance induced by a high-fat diet can be reverted by exercise training in mouse skeletal muscle^96^. The authors quantified ~3000 proteins by HILIC fractionation followed by DDA-MS, including MUP1 as an AMPK substrate regulating the increase in insulin sensitivity following exercise training. Batista et al. derived induced-pluripotent stem cell (iPSC) lines from T2D patients and matched controls followed by extensive phosphoproteomics following differentiation^97,98^. Their powerful approach allowed the rapid and simultaneous manipulation/stimulation of cells in a paired manner independent of systemic endocrine hormones and metabolites. The authors characterised thousands of phosphorylation sites revealing prominent deregulation in the non-canonical insulin signalling pathways involving nuclear proteins that mediate transcription and splicing. These surprisingly findings suggest that the development of diabetes could be independent of systemic factors, however, these findings have yet to be confirmed in vivo. Another important limitation of these studies is that a comprehensive paired proteomic analysis was absent, and thus, differences in phosphopeptide abundance could be driven by changes in protein abundance rather than phosphorylation state. Finally, analysing shared changes in response to two or more conditions (e.g., diet, insulin, exercise training) is another powerful approach to elucidate mechanisms of important biological processes.

Recently, these limitation have been elegantly addressed by Kjærgaard et al., who analysed the signalling pathways in human skeletal muscle activated by either exercise and insulin stimulation (both of which promote glucose uptake), in the same individual^99^. Utilising two batches of 11-TMT plex, each with a pooled internal reference to normalise across the batches, the authors preformed phosphoproteomics in a paired, cross-over design by high pH (HpH) fraction DDA-MS. The results identified the phosphorylation of S709 on REPS1 that was increased under both conditions, which was attenuated in the skeletal muscle of both insulin resistant mice, and in biopsies from individuals with T2D. The authors also validated its function by showing that knockdown reduced insulin-stimulated glucose uptake in vitro. In a similar fashion, Needham et al. analysed skeletal muscle biopsies from individuals with or without insulin resistance, before and after exercise, and following a 4 h washout period. Subsequent biopsies were also taken after insulin stimulation by a hyperinsulinemic-euglycemic clamp^100^. An exceptional advantage of their experimental design is the one-legged exercise intervention approach which allowed pair-wise analysis of the exercise leg to the rested leg to understand how exercise confers improvements in insulin sensitivity. Moreover, the use of individuals with a spectrum of insulin resistance alleviates stratification of participants into groups and leverages individual variation which provides greater power to associate phenotypic differences. As a result, the authors identified mTORC1-dependent phosphorylation of S377 on AMPKα2 as a core substrate that is defective in insulin resistance, and S441 on MINDY1 as a protein associated with increases in insulin sensitivity after exercise. The authors also validated that knockdown of MINDY1 potentiated insulin-stimulated glucose uptake in vitro.

Most recently, Kjærgaard et al. presented one of largest phosphoproteomic cohorts with the analysis of >120 subjects with varying degrees of insulin sensitivity, from euglycemic to diabetic participants^101^. Proteomics and phosphoproteomics was performed before and after insulin stimulation, and similar to the work by Needham et al., associations were performed to various paired phenotypes, including glucose uptake. Their work identified ~184 phosphosites that were associated with insulin sensitivity, leading to the similar identification of S377 on AMPKα2. Interestingly, S65 on the AMPKγ3, a human-specific site, was the most negatively correlated with insulin sensitivity, emphasising the importance of studying samples of human origin. Moreover, the authors reveal that the fasting/non-insulin stimulated phosphproteome is a critical determinant for whole body insulin sensitivity and challenges the preconception of the field which primarily focus on the feeding/insulin stimulated phosphoproteome.

At this point and pertinent to this review on the technical aspects of skeletal muscle proteomics, it is worth mentioning that the latest large-scale studies by Needham and co-workers, and Kjærgaard and colleagues utilised single-shot label-free phosphoproteomics. In the case of the latter study by Needham and colleagues, >26,000 phosphopeptides were identified in total, and >30,000 identified in the latter study by Kjærgaard and colleagues which is outstanding coverage given the complexity of skeletal muscle. However, upon filtering, ~13,000 phosphopeptides were identified by Needham and co-workers in at least 50% of the cohort which they used for statistical analysis, and this number dropped to only ~4000 phosphopeptides when filtering for phosphopeptides identified in every participant. For data acquired by Kjærgaard and colleagues, ~31,000 phosphopeptides were identified in at least 25% of the cohort which they used for statistical analysis, and this number dropped to~8200 when filtering for phosphopeptides identified in every participant. These data show that despite analysing the samples with DIA, comprehensive coverage of human muscle cohorts is still a hurdle. Missing data has important ramifications for studies employing paired experimental designs where multiple subject datapoints are required for optimal analysis. It is likely that the level of missing data is more pronounced in human muscle biopsies which have higher genetic and environmental diversity compared to e.g inbred mice, and this is further exacerbated by the higher dynamic range and complexity compared to e.g isolated cells.

Although not the focus of this review, the choice of statistical approaches is another critical factor in clinical muscle proteomics, profoundly shaping results and their interpretation. For instance, Needham et al. identify 512 insulin-stimulated glucose-uptake-associated phosphosites in 19 individuals, while Kjærgaard et al. report 184 sites in their discovery cohort of 77 individuals. The focus should not solely be on increasing the total number of identified/significantly different proteins or phosphosites. Instead, the emphasis must shift towards identifying high-confidence, prioritized candidates that can be pursued in downstream functional studies. Achieving this requires a more standardized consensus on statistical models and robust methods for *p*-value correction to ensure reproducibility and comparability across studies. Addressing such challenges is especially critical given the inherent heterogeneity of human skeletal muscle biology, which necessitates scaling up proteomics and phosphoproteomics studies to include significantly larger cohorts. This expansion should be accompanied by rigorous and reproducible sample preparation workflows, whether utilizing TMT-based quantification or label-free mass spectrometry. Scaling and standardization together will be crucial in overcoming the challenges posed by the complexity and diversity of human muscle biology.

Finally, and most importantly, while great strides have recently been made to map phosphoproteomic landscapes across mouse models^102^ and the above studies in humans, none of them definitely prove that phosphorylation of the identified associations are causal in promoting skeletal muscle glucose uptake or the exercise-mediated improvements in insulin sensitivity. The field needs to shift to the characterisation of functional phosphorylation events e.g. via mutagenesis combined with physiological assessments. Computational approaches have provided clues^103^ and combined with high throughput screening^104^, functional characterisation of signaling is expanding. Integrating protein and post-translational modification (PTM)-quantitative trait loci (QTL) of skeletal muscle function and disease traits combined with additional statistical approaches such as mediation analysis may provide alternative avenues to identifying causal drivers of skeletal muscle metabolic health^20,105^.

## Perspective

Over the last 2-3 decades, improvement in sample handling techniques, development of more sensitive mass spectrometers and novel experimental approaches has propelled the field enormously, enabling characterisation of many facets of the skeletal muscle proteome. Looking ahead, as the field leverages the latest advancements in artificial intelligence, multi-omic and spatial technologies, we can only begin to imagine the possibilities and questions that can be addressed. Here, we discuss the future perspectives of the field and applications that most excite us.

Spatial proteomics/deep visual proteomics (DVP)^106^ was recently named “Method of the Year: 2024” by Nature Methods^107^. These latest advancements pioneered by the Mann group, has allowed seamless integration of sub-cellular resolution images with ultra-high sensitivity mass spectrometry to create spatial proteomics maps. The use of artificial intelligence driven single cell segmentation coupled to automated dissection and single cell-MS has bypassed traditional limitations (i.e., imagine mass cytometry), by allowing near complete coverage of the proteome. Even more excitingly, these data set could then be further complimented by spatial transcriptomic or multi-omic data to understand the regulation of gene expression and protein abundance. Furthermore, the ability to monitor protein turnover rates using pulsed stable isotope labelling at the single-cell level is expanding and likely to provide important information on protein function and cellular heterogeneity^108^. Overall, applications of DVP to understand how fibre/proteome heterogeneity in muscle fibres and other muscle cell types such as MuSC, can be selectively impacted by settings of health and disease could provide exciting new treatment avenues.

Another attractive approach is to combine proteomics with other omic technologies, such as RNA sequencing. Single-nuclei RNA sequencing (snRNAseq) is an alternative approach to single-cell RNAseq (scRNAseq) that provides ultra-deep transcriptome profiling. The use of snRNAseq is especially necessary for skeletal muscles due to the presence of multi-nucleated cells which cannot be isolated/profiled using traditional scRNAseq means. However, profiling the nuclei and its surrounding proteome have proved difficult. Thankfully, recently advancements, such as the development of proximity labelling-based MS, have enabled enrichment of previously inaccessible subcellular niches. All in all, combing snRNAseq with proximity labelling based profiling of the transcriptome and proteome of subcellular compartments, which are driven by different subcellular nuclei populations, could be a very powerful way to understand shared signalling pathways important for health and disease.

Finally, developments in autonomous sample preparation by liquid handlers, coupled to the latest developments in LC, including Vanquish by Thermo and the Evosep One^68^, has further reduced sample handling steps, increased retention time precision and sample throughput. Moreover, advancements in sample separation technique and mass analysers have increased the ability profile the proteome to an even greater extent. For example, the latest hybrid Orbitrap-Astral MS by Thermo Scientific and the Trapped ion mobility separation coupled to Time of Flight (TimsTOF) MS by Bruker has the capability to identify >10,000 protein groups from human cell lines in less than 30 min of runtime. Further improvements in: (i) acquisition speeds (>300 Hz), (ii) online liquid-phase or gas-phase separation, (iii) intrascan dynamic range, and (iv) parallel acquisition or fast orthogonal peptide fragmentation on hybrid instruments are likely to see further improvements for analysis of complex tissue samples like skeletal muscle. A further consideration is that greater sequence coverage in bottom-up proteomics to increase the number of peptides quantified per protein combined with improved signal-to-noise detection will increase both precision and accuracy particularly for low abundant proteins. Finally, improvements in latest proteogenomics workflows [PMID: 36959352] applied to clinical analysis of human skeletal muscle are likely to see personalised proteoform analysis including detection of alternative start/stop sites, splicing variants, single nucleotide polymorphisms and peptides translated from unannotated regions of the genome. We can only begin to imagine the boundaries that new discoveries and technology will push in allowing the identification of high confident proteins relevant to health and disease.

As the field head towards a new era of proteomics research, we feel humbled and honoured to be part of this community and are excited to what the future holds. One day, the collective effort of the community will finally unravel the mystery of the skeletal muscle in the larger context of health, disease and physiology.

## Data Availability

Raw proteomics data generated in this manuscript are available upon request.
